# Patients’ preferences of cutaneous leishmaniasis treatment outcomes: Findings from an international qualitative study

**DOI:** 10.1371/journal.pntd.0007996

**Published:** 2020-02-24

**Authors:** Astrid C. Erber, Byron Arana, Afif Ben Salah, Issam Bennis, Aicha Boukthir, María del Mar Castro Noriega, Mamoudou Cissé, Gláucia Fernandes Cota, Farhad Handjani, Liliana López-Carvajal, Kevin Marsh, Dalila Martínez Medina, Emma Plugge, Trudie Lang, Piero Olliaro

**Affiliations:** 1 Nuffield Department of Medicine, Centre for Tropical Medicine and Global Health, University of Oxford, Oxford, United Kingdom; 2 Department of Epidemiology, Center for Public Health, Medical University of Vienna, Vienna, Austria; 3 Drugs for Neglected Diseases Initiative (DND*i*), Geneva, Switzerland; 4 Institut Pasteur de Tunis, Tunis, Tunisia; 5 Department of Family and Community Medicine, College of Medicine and Medical Sciences, Arabian Gulf University, Manama, Bahrain; 6 National School of Public Health, Rabat, Morocco; 7 Department of Public Health, Institute of Tropical Medicine, Antwerp, Belgium; 8 Centro Internacional de Entrenamiento de Investigaciones Médicas (CIDEIM), Cali, Colombia; 9 Universidad Icesi, Cali, Colombia; 10 Centre MURAZ, Bobo-Dioulasso, Burkina Faso; 11 Instituto René Rachou (IRR), Fundação Oswaldo Cruz (FIOCRUZ), Minas Gerais, Brazil; 12 Molecular Dermatology Research Center, Shiraz University of Medical Sciences, Shiraz, Iran; 13 Programa de Estudio y Control de Enfermedades Tropicales (PECET), Universidad de Antioquia, Medellín, Colombia; 14 Facultad de Medicina “Alberto Hurtado”, Universidad Peruana Cayetano Heredia, Lima, Perú; 15 Departamento de Enfermedades Infecciosas, Dermatológicas y Tropicales, Hospital Cayetano Heredia, Lima, Perú; 16 UK Collaborating Centre for the WHO Health in Prisons Programme, Public Health England, Reading, United Kingdom; 17 Special Programme for Research & Training in Tropical Diseases (WHO/TDR), Geneva, Switzerland; University of York, UNITED KINGDOM

## Abstract

**Background:**

Cutaneous leishmaniasis (CL) is a disease that often affects exposed skin areas and may heal leaving lifelong scars. Patients’ expectations from treatment are rarely considered in drug development for CL. An initiative aiming to address shortcomings in clinical trial design and conduct for CL treatments involving the researchers’ community is on-going. This manuscript presents patient-preferred outcomes for CL and an assessment on how to consider these in the conduct of future trials.

**Methodology/Principal findings:**

We report preferred treatment outcomes by 74 patients with confirmed CL in endemic regions of Brazil, Burkina Faso, Colombia, Iran, Morocco, Peru and Tunisia during individual in-depth interviews. Beyond outcomes customarily considered in trials (such as lesion appearance and adverse events), patients talked about a large number of outcomes related to quality of life, such as pain, scar formation, and others affecting their work and daily activities. They also reported fears around getting rid of the parasite, disease recurrence, and possible sequelae.

**Conclusions/Significance:**

The study results provide a rich insight into important outcomes for CL treatments, as well as related topics, from the perspective of a diverse patient population. Among the outcomes identified, we argue that those related to quality of life as well as recurrence should be included to a greater extent for assessment in clinical trials, and discuss the suitability of measurement instruments such as the Dermatology Quality of Life Index (DLQI). Interviews also point out the potential need to address concerns related to parasitological cure or scar formation, such as social stigmatization and disability. In addition, patients should be given information in order to clarify reported misconceptions. This study therefore suggests a methodology for consulting CL patients on outcomes as elements of clinical trial design, and how to incorporate these outcomes in trials. It also discusses how reported outcomes could be addressed in clinical care.

## Author summary

Cutaneous Leishmaniasis (CL) is a parasitic disease transmitted by sand fly vectors causing lesions and potentially life-long scars. Trials for CL treatments so far have not included patients’ input, such as their preferences on outcomes. This study reports treatment outcomes seen as important by patients that were explored through semi-structured interviews conducted in CL-endemic regions in Brazil, Burkina Faso, Colombia, Iran, Morocco, Peru and Tunisia. In addition to outcomes mostly considered in trials centered on lesion evolution and adverse events, patients reported a wide range of further outcomes and concerns. These are related to their quality of life, but also parasitological cure, potential for recurrence and sequelae of the disease. We suggest possibilities on how to include and measure a number of these patient-preferred outcomes into trials, and discuss ways of considering others in the context of treatment and clinical trials, e.g. *via* information provided to patients.

## Introduction

Cutaneous leishmaniasis (CL) is the most frequent form of leishmaniasis, one of the Neglected Tropical Diseases (NTD) [1]. CL occurs across tropical, subtropical and temperate regions and is broadly divided in old-world and the new-world forms (OWCL and NWCL), which are caused by different species and cause a range of clinical manifestations [2]. It causes visible lesions on exposed parts of the body, which can be distressing, discomforting and potentially leave life-long scars, although not life-threatening. At present, there is no treatment which is effective, safe and easy to administer against all clinical forms of the disease. This is largely due to the lack of investments in drug development for a disease that affects essentially impoverished populations, it is also contributed to by weaknesses in the design and conduct of clinical trials for CL interventions which render meta-analysis of results and evidence-based recommendations difficult [2–9].

Progress has been made in addressing these shortcomings through reaching consensus across the OWCL and NWCL researchers’ community on critical aspects of clinical trial methodologies for CL [10,11], however, patients’ views have not yet been genuinely considered, especially regarding treatment outcomes. Trials conducted so far emphasize clinical and parasitological endpoints, but rarely report outcomes affecting patients’ quality of life (QoL), or factor in patients’ needs in the design phase: A recent systematic review on outcomes included in trials for CL interventions [7] found that nearly all (100% and 86.35% of the studies which included initial and definitive cure, respectively) used complete re-epithelisation of the ulcer as the definition of ‘cure’. Additional criteria that were variably present included: absence of active lesion, no relapse, negative parasitological test, clinical improvement, no appearance of new lesions, and/or reversible hypopigmentation. Additional information can be derived from previous systematic reviews: of the 48 studies included in systematic reviews of trials conducted on NWCL, seven did not report adverse events, and none measured the degree of functional and aesthetic impairment, prevention of scarring, or QoL [3,8]. Among 89 trials considered in a Cochrane Collaboration systematic review for OWCL, prevention of scarring was measured in eight studies, and adverse events in all but eight; no study was found to measure the degree of functional or aesthetic impairment, or QoL [6].

There is increasing consensus that patients’ involvement in health research can lead to higher rates of enrolment and retention, and improve its translation into clinical practice [12]; it meets also an ethical mandate towards ‘democratizing‘ the research process [13]. Involving patients in the definition of treatment outcomes is recommended, though not frequent practice yet [14–16], but has proved useful in identifying outcomes not previously brought up by other stakeholders [17,18]. The COMET (Core Outcomes Measures in Effectiveness Trials) initiative [19] is committed to the development of Core Outcome Sets (COS), actively involving patients in their development. A COS, consisting of Core Outcomes or Core Outcome Measures (COMs), is an agreed minimum set of outcomes that should be measured and reported in clinical trials of a specific disease or trial population [14,17,18,20].

The purpose of this study is to present findings relating to OWCL and NWCL patients’ expectations on desired treatment outcomes as part of a large multi-centre qualitative study exploring patients’ perceptions and understanding of CL. This will complement the consensus reached among the clinical research communities [10,11]. Together, these comprehensive approaches are intended to improve the design of interventions and the way these are assessed, so as to achieve better care for neglected populations affected by CL.

## Methods

We conducted 74 individual, semi-structured in-depth interviews with CL patients at sites in seven endemic countries (Brazil, Burkina Faso, two regions in Colombia, Iran, Morocco, Peru, and Tunisia), as described in the protocol [21]. These countries were chosen due to their CL disease burden, prevalent *Leishmania* species, and accessibility for research. Interviews were conducted by the local principal investigators (PIs) in the endemic regions, who are researchers or health care professionals (HCPs) working with CL patients.

All sites used collaboratively-developed, equivalent protocols and interview topic guides ([21], supplementary file 1), with minor adaptations depending on the country specific ethics committees’ requirements. For this study, a two-step sampling approach was used. Maximum variation was sought within a defined patient spectrum along characteristics such as patients’ gender, age, treatment status (before, during, after treatment), clinical lesion presentation and causative *Leishmania* species, under the assumption that this would cover the range of patients’/disease profiles. The background parameters informing the sampling strategy, including CL epidemiology at each site, can be found in [21], supplementary file 2.

The interviews lasted about one hour, were conducted in the patient’s mother tongue and audio recorded; interviewers took notes before, during and after the interviews. Each recording was transcribed verbatim into the language in which the interview was conducted and then translated into English for analysis, either by the PIs or a dedicated person. Translated transcripts were verified and if necessary back-translated by the PIs who conducted the interviews, in order to confirm the accuracy of translation, before they were forwarded for analysis. Detailed in-text notes, e.g. explaining regional dialect or colloquial expressions, and communication in person to clarify any misunderstandings helped with analysis and interpretation.

Thematic content analysis with a coding framework following the six distinct steps described by Braun and Clarke [22] was used. The coding framework was developed collaboratively during a face-to-face meeting of investigators after familiarization with the data, based on three initial interview transcripts from Morocco. Transcripts were read twice, and sections organized under different, suitable headings. Headings were grouped into seven broader domains (nodes): *Costs*, *Symptoms*, *Impact on daily life*, *Relationship with health system*, *Treatment*, *Knowledge and understanding of disease*, and *Diagnosis* (in no particular order), as well as suitable sub-nodes. Consensus was achieved through discussion among the investigators.

**Fig 1:**
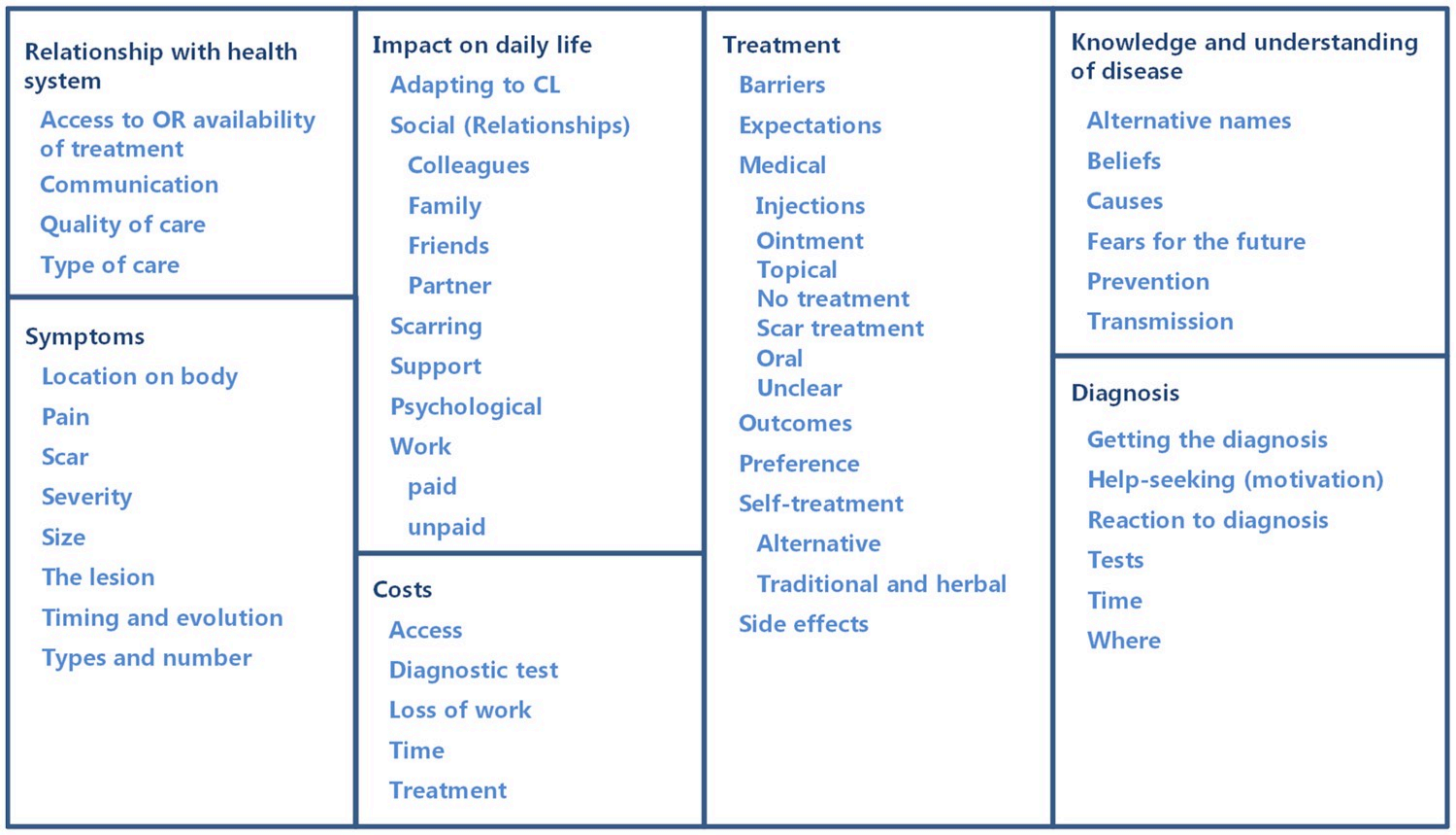
NVivo coding framework for interview transcripts. This framework was established for coding interviews. Nodes are depicted in dark blue, sub-nodes in blue.

For identifying outcomes, data in all transcripts were coded to a subset of nodes including *Outcomes*, *Expectations* and *Preferences* (in the category *Treatment*), as well as *Scar* (in the category *Symptoms*), *Medical* and *Self-treatment* as well as *Adverse events*. This allowed us to capture contextual information) using NVivo 11. During analysis, the coding framework was subject to continuous updating, with the final framework shown in Fig 1, reflecting the rich information found in the interviews.

Themes were then identified using pen-and-paper due to the high volume of data: Transcript sections that had been coded to relevant nodes were printed from NVivo and manually arranged into key themes (corresponding to outcome domains) and subthemes (corresponding to outcomes). Examples for themes emerging include social stigmatization, or fear of sequelae. Themes are concepts or propositions that describe, help to interpret and explain aspects of the data. They were articulated and developed by comparison between and within interviews by two researchers (ACE and EP), specifically looking for emerging patterns, similarities and differences [23].

Subsequently, all outcomes reported as relevant by patients were assessed for possible inclusion into clinical trials based on (a) whether they were clinically meaningful and measurable by the research group, based on published literature, and (b) whether they could be addressed by additional or alternative measures. Outcomes were grouped over several steps by exclusion: (1) Outcomes that were found to be already incorporated and reported in clinical trials. (2) Outcomes based on unfounded fears or misconceptions related to CL. (3) Outcomes that were considered meaningful and measureable and could be included in clinical trials. (4) Outcomes with a longer-term impact, those that were experienced due to comorbidities, and outcomes having gender-specific and cultural connotations surrounding the broader psychological and social impact of scar formation.

### Ethics statement

Ethical clearance of the protocol was obtained from the following institutional review boards (IRBs) and ethics committees (ECs) responsible for the entire study, and the respective sites: World Health Organization Research Ethics Review Committee (WHO ERC), Geneva, Switzerland, Comité d'éthique de la Faculté de Médecine et du CHU Hassan II Fes, Fez, Morocco. Comité d'éthique Biomédicale de l'Institut Pasteur de Tunis, Institut Pasteur de Tunis, Tunis, Tunisia. Comité Institucional de Ética de Investigación en Humanos (CIEIH), Centro Internacional de Entrenamiento e Investigaciones Médicas (CIDEIM), Cali, Colombia. Comité d'éthique institutionnel du Centre MURAZ, Bobo-Dioulasso, Burkina Faso. Instituto Rachou (IRR), Fundação Oswaldo Cruz (FIOCRUZ), Minas Gerais, Brazil, and Comissão Nacional de Ética em Pesquisa—CONEP, Brazilia, Brazil. Shiraz University of Medical Sciences (SUMS) Ethics in Research Committee, Shiraz, Iran. Comité de Bioética de Investigación en Humanos, Sede de Investigación Universitaria (CBEIH-SIU) of the University of Antioquia, Medellin, Colombia. Comité Institucional de Etica para Humanos, Universidad Peruana Cayetano Heredia (CIEH—UPCH), Lima, Perú. Oxford Tropical Research Committee (OxTREC), University of Oxford, Oxford, UK.

Only patients above the age of consent were interviewed. Participants gave their consent by signature, or an appropriate alternative as specified by the relevant IRBs, to the interviewer or a designated person using an informed consent declaration. To obtain consent from participants who were unable to sign, at least one literate witness was chosen, whenever possible by the participants themselves, from outside the research team.

Ethical procedures at each site observed the respective IRB guidance, and are further detailed in the study protocol [21].

## Results

### Patients’ characteristics

The characteristics of the interviewed patients are listed in S 1 File. Sixty-two percent (n = 46) of participants were male (reflecting an estimated distribution of 60% men and 40% women overall at the sites [21]). At each site, the distribution was also in line with previous estimates of gender distribution among patients in the study area [21]. The median age of patients was 35 years (range 18–73 years). There were equal numbers of OWCL and NWCL patients. Patients live in areas where the predominant *Leishmania* species are L. *(Viannia) braziliensis*, *peruviana*, and *panamensis* for NWCL and L. *major* and L. *tropica* for OWCL. On average, patients had 3.4 (SD = 6.6, median = 2) lesions, with considerable variation in total (Q1 = 1, Q3 = 3.75, IQR = 2.75) and across sites, with the median numbers ranging from 1 to 6. Five percent of patients have had CL diagnosed but not started treatment, 47% were under treatment, 46% had completed their treatment and 1% had remained untreated; those receiving only symptomatic (medical) treatment were also considered as being treated.

### Outcomes relevant for patients

**Fig 2:**
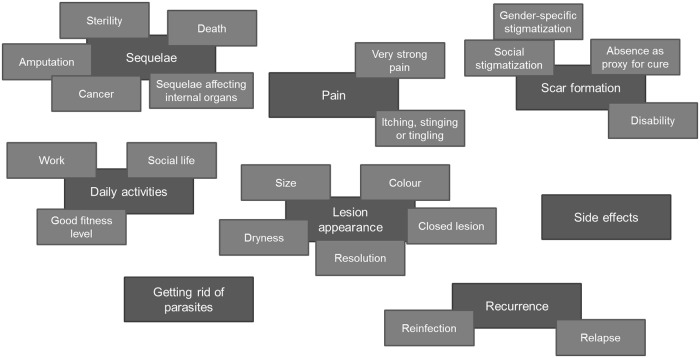
Key themes and subthemes of outcomes relevant for patients.

Eight broad themes related to outcomes, corresponding to what patients would like to see or expect from treatment, and what they would not like to experience, emerged from the data. These were: *Sequelae*, *Pain*, *Scar formation*, *Daily activities*, *Lesion appearance*, *Getting rid of parasites*, *Relapse and reinfection* and *Side effects* (Fig 2). These domains are comprised of subthemes which correspond to individual outcomes. The wording corresponds to medical terminology in line with researchers’ background and the study aims, and importantly, facilitating comparison with the literature and integration into an outcomes classification [19]. We avoided prioritizing certain outcomes over others; therefore we did not conduct frequency analysis and avoided to present them in a particular order, in line with the exploratory nature of the study.

Patients’ unique identifiers contain a two-letter code corresponding to the site where they were interviewed (BR-Brazil, BF-Burkina Faso, CC-Colombia/CIDEIM, CP-Colombia/PECET, IR-Iran, MO-Morocco, PE-Peru, TU-Tunisia). For two quotes, interviewer’s questions (I) were left in as they were deemed necessary for comprehension of the patients’ responses (R).

### Sequelae

Patients saw successful treatment as avoiding sequelae such as large lesions leading to amputation of limbs, disease progression to affect internal organs, cancer and even death. If not successfully treated, several patients feared amputation of limbs as potential complication, such as this young man from Brazil:

[…] For me it is a bad bug starving, it keeps eating, eating and eating. […] The injury will go deeper and it can take a nerve, one may lose the movement and may need to have to amputate a limb!

(BR05)

For a diabetic young woman from Tunisia, her fear of amputation of a leg due to CL was so strong that she reported suicidal thoughts:

*A catastrophe […] in my life named leishmaniasis, I swear. I can’t talk. I’m thinking how my leg could be amputated. How my life will look like after amputating my leg, I think about suicide, at the same time I think about my daughter how she will calm down after my death. I can’t live without my leg*.

(TU04)

Several patients mentioned the possibility of the disease affecting internal organs and cancer as a sequel of CL.

*I think that cure would be the decontamination of the infection. The most important thing is the elimination of the risk of compromising the internal organs*.

(BR09)

*(…) It will change to a cancer that what he [someone at the health centre] said. […] He said to me it is possible to change […] with the years and he said I had a lucky chance to not get the disease inside me. This form of disease is the most dangerous one*.

(MO08)

Several patients feared death as a potential consequence of CL; a female patient from Burkina Faso was afraid that CL could kill her—a CL outbreak erupted there in 2013. The patient came from a previously unaffected area where the population was unaware of the disease.

A number of male patients in Colombia being treated with pentavalent antimonials and pentamidine isethionate as first- or second-line treatment, respectively, talked about their fears that CL treatment could leave them sterile, that is, unable to father children. Patients referred to rumours they have heard, or someone telling them about it.

*Many people say you cannot have children. So that could be it. […] Those are comments you hear. From that to reality, I’d say there is a 20% chance of that to happen. So that doesn’t affect me at all. […] Friends always say that: You have children already? No. Oh boy, you lost your chance now. [laughs] I don’t pay attention to them though*.

(CP10)

### Pain and unpleasant sensations

Itching, stinging or tickling sensations or unspecified pain was mentioned by patients as symptoms. Unspecified, very strong pain was mentioned several times, and most notably by the majority of patients in Burkina Faso. Patients described it differently: One male patient compared it to chili pepper being put onto the lesion, or a female patient experienced the pain associated with her CL lesions as ‘being burnt by fire’, rendering her unable to work.

*I went to the health centre and they told me that there is no treatment for this disease. Lesions became painful and I went to health centre again and they told me that there is no treatment for this disease. Lesions were painful and itching. […] I didn’t work in my farm because of the pain that is like being burnt by fire. I didn’t get anything in that year from my farm*.

(BF02)

In contrast, other patients emphasized that CL itself was painless. By them, pain was experienced as treatment side effect:

*[…] Because as I’d told you, the illness does not cause any pain, but the treatment itself does*.

(CC01)

### Daily activities

Patients mentioned that being cured for them means being able to perform work, to have a normal social life without their family being affected, and a good fitness level. Being able to go back to work, either paid or by supporting family, was seen as associated with being healthy again. This male patient in Burkina Faso reported being unable to work while sick, thereby decreasing his income to an extent that he was unable to provide for his family:

*[Healing] means that you got a good recovery and your family is no longer suffering and your kids too*.

(BF09)

For several patients, to be able to go out and have a normal social life, free from stigmatization, was a very important component of being healed.

*In the future I hope I will have a happy life, to be an active person, to go out and to communicate with others*.

(TU03)

### Lesion appearance

Different components of the lesion appearance were considered as important outcomes of treatment, mostly whether the lesion was closed, its dryness, its size, and its colour, and often a combination of these elements. A closed lesion corresponds to re-epithelialization in medical terms, that is, the wound being covered with an intact tissue.

*[Healing means] let the wound close, heal. […] Covered, that’s what I imagine*.

(PE05)

For several patients, a dried wound and a reduction in wound size indicated cure. Others expected treatment to remove the discoloration.

*[After treatment I expect the lesions] at least to return to the same skin colour, right? Because I see it all bruised*.

(PE03)

Often, patients mentioned ‘normal skin’ as an outcome, but did not specify it further.

*I guess it will disappear totally. I will not see any knob on it and my skin will return to normal*.

(MO13)

### Getting rid of the parasite

The absence of the pathogen, and, importantly, confirmation by a HCP were seen as a relevant outcome by many patients. The pathogen was in many cases specified as a parasite.

*Cured, well, to know that the parasite is eliminated from the body a hundred percent, basically. Regardless of the scars left. That is the least of my worries. That will be something one can handle as time goes by. [So my main concern is] that there aren’t any creatures in my body*.

(CC01)

### Recurrence

Fear of recurrence, that is, relapse and reinfection, was an issue for many patients. In most cases patients were clearly able to distinguish between relapse, with the lesions occurring in the same skin area, and reinfection by a sand fly, in particular if they went back to the endemic areas.

This patient from Burkina Faso, talks about his concerns on the community level as well as on an individual level. He is afraid that his scars could turn into active lesions again:

*Oh! I pray [to] God so that this disease never comes [back again]. […] This disease has appeared suddenly and has disappeared now. It could come back again. […] I want God to eradicate definitely this disease here [in this village] so that people can be in good health and then this disease can’t come back again. […] I can say that it is not still present. My lesions have been healed but I am afraid that the scars become lesions*.

(BF04)

Patients felt very concerned and uncertain about a relapse. Some specifically mentioned that they did not feel cured as long as there was this possibility.

*I consider it [late complications and relapse] and it makes me very worried. […] According to the search I performed, future lesions can appear and mucosal lesions as well. My biggest concern is that today: the disease relapse. It is very important. […] and, about the risk of future lesions, we can’t even know. […] I have no idea whether I’m cured or not*.

(BR03)

Often, patients were aware that they could be reinfected despite a successful treatment, and were afraid of it happening. In particular the soldiers interviewed in Colombia were well informed about the danger of reinfection if they went back to the endemic regions, and often mention having seen colleagues in the army getting infected multiple times.

An elderly male patient from Iran discusses possible immunity to reinfection after having healed by itself, after around one year. This is also reflected in the traditional name *Salak* (in Persian *Sal* means *year*) given to CL:

*Well according to experiences, the reason that they named it Salak that it lasts one year and then it will get healed. […] It will get healed and then one’s body is immune to CL. […] As I said, I heard this from people*.

(IR03)

### Scar formation

Scars were important and the absence of a scar was seen as a sign of being healed (as a proxy for cure). If in visible areas, many patients expressed their fear of social stigmatization. Many reported being affected by social stigmatization with a visible scar, ranging from embarrassment about others asking about its origin, to being ridiculed. Others reported fears of not finding employment or a suitable partner with a visible scar. Several participants emphasized gender-specific aspects, for example the problems a visible scar in the face could pose for unmarried women.

This Brazilian woman mentions and reiterates that a visible scar might indicate that she is not yet fully cured:

*I think that if this scar disappeared, then I would have the notion that it is healed. The scar being here makes me remember of this disease. […] I think when you see the wound, when you see the scar, you’re remembering. Sometimes, this question arises… Will it be gone? It is insecure*.

(BR03)

Some patients, predominantly men, did not see their scars as problematic.

*I don’t care much [about the remaining scars]. I have already some. Look at this. […] One day I put a cup of gunpowder in the fire and it exploded in my hand. I’m not worried about scars. There is a good cream for scars anyways*.

(CP10)

*No, they are completely healed but the scar remained. The most important thing that it is over, I don’t care about the scar. I’ll marry another woman! I'll undress my shoulders, I don’t care*.

(TU02)

In some regions patients freely talk about the availability of scar treatments, for example in Iran and Tunisia, where cosmetic surgery seems to be common as it is mentioned frequently in the interviews. However, whereas CL treatment is free, the costs for scar treatment are not covered by insurance, and pose a financial problem to many patients. Several patients pointed out how a scar affects girls or women more than men, specifically if the scars are located on their face or other visible areas and if the girl is still unmarried.

This young man from Morocco talks about the problem that scars pose for young women. He points out psychological problems due to deformations, and compares the CL scar to deformed teeth:

*She will have a serious problem which can lead to psychological complexes in her life. […] She can become psychologically sick because of the scar, I know people who have a problem by The Will of God. For example, a girl with deformed teeth and defected look, it makes her psychically sick, although for the teeth there is a solution because the medical science became now more developed*.

(MO02)

He continues explaining the impact he thinks his scar will have on his own relationships with women, and in his workplace:

*[Should I meet a girl], I can convince her by giving details that it is a disease and [that] I followed the treatment prescribed by the doctor, to not be scared any more. […] I: If the scar will remain, you may have any problem in your work? R: I don’t understand, do you mean I must keep my basketball cap whole working day to hide the scar? No, I can’t do it all day long*.

(MO02)

Most notably, a woman from Iran, with three scars on her face and hands, told her story, and how has severely affected her social life and two marriages:

*I was 5 years old when I got CL and didn’t know what it was. […] All the other children had it on their hands or feet. Me and my sister had it on our face… […] Well I got married at 17 and I got divorced because of this issue. He told me something about my face and I finally got separated when we were engaged. […] I got married again, and my husband’s skin is white without any spots. I had to use creams since I was a kid. […] Yeah, I married twice and I didn’t have a wedding ceremony both times due to my CL. […] Because they took films at weddings and the camera takes a close shot from your face and I didn’t want that… […] As much as I remember, I put on makeup all the time […] It’s been 4.5–5 years that I married my current husband… emmm… I think yesterday was the first time that he saw me without makeup [pause] I feel ashamed to look in his face… I looked down. […] I want to go outside but without makeup*.

(IR08)

By contrast, this Peruvian patient explains neither being ashamed, nor being afraid to be stigmatized in his social environment:

*I: And, do you mind that scar? R: No doctor, normal, [it doesn’t matter] in my work. I: And, what about the one on your face? R: The same. I: And is there no problem? R: No, no problem. What can I do doctor? I: Don’t you mind? Are you not ashamed? R: No, no. I: Some people feel ashamed; they do not want to go out. R: But, since I was a boy, and after all in my land, all my countrymen know me, and we know each other, what can I do? It will be the same*.

(PE07)

Some patients feared that the scar would result in a permanent disability, such as a young male soldier from Colombia who suffered a lot from its location very close to his eye, and was afraid that the complications and pain would continue with a scar resulting from that wound.

### Side effects

For this specific theme, the analysis focusses on fears and conceptions of patients. An overview of the treatments administered can be found in [21], supplementary file 2. Common side effects reported were headaches, local swelling, pain (e.g. back or stomach ache, pain in the joints, muscles, kidneys, legs or the testicles), loss of appetite, fever, tiredness and dizziness. Patients also complained about palpitations, shivers, weakness, weight loss and vomiting to a lesser extent. These adverse events are documented in the literature [3,4,6,24]. In general, people felt informed about adverse events related to the treatment.

*I've heard it’s to check the organs to see if they are in good shape; because the Glucantime, it’s so heavy that it affects the heart, kidneys, liver …It affects pretty much every organ, so you must have them in good shape. […] I've seen partners who have two and three treatments already and it’s not the same, doctor. I've seen them having troubles with their kidneys and how that decays their bodies. I've seen people that with the Glucantime only they lose a lot of weight*.

(CP02)

Pain was described as a common side effect of treatment, sometimes perceived as unbearable:

*Yes, it was very hurtful. When you have already received 5 of the 10 injections, the medicine doesn’t go through because the buttocks are so swollen that they are forced to suspend the treatment. You cannot endure the pain. I was one of those. I went to therapy but it wasn’t enough. I was all the time lying in bed. I couldn’t even walk. Very painful*.

(CP04)

## Discussion

### Summary of findings

Patients have clear ideas about desired treatment outcomes. These relate to their personal health and wellbeing as well as their social and socioeconomic context, and comprise the domains *Lesion appearance*, *Side effects*, *Recurrence*, *Getting rid of the parasite*, *Scar formation*, *Daily activities*, *Pain* and *Sequelae*. Many of the reported outcomes are generally not reflected in trials conducted to date, which focus mostly on outcomes looking at lesion and infection indicators (such as absence of an active lesion, complete re-epithelisation, parasitological cure, no appearance of new lesions and/or reversible hypopigmentation) [3–8].

Patients’ fears surrounding persistence of the parasite and disease relapse are supported by available evidence. Several forms of leishmaniasis can occur after healing: Leishmaniasis recidivans is a chronic form of CL due to *L*. *tropica* or *L*. *(Viannia) braziliensis* occurring 1–2 years after healing of the initial lesion, usually in the face, causing microsatellite and confluent lesions that finally ulcerate at the border of the initial scars. Mucocutaneous leishmaniasis (MCL), caused by *L*. *(Viannia) braziliensis* and other NWCL species, affects nasal and oral mucosa, and evolves after cure of CL and a variable period of latency. It potentially leads to disfiguring mutilations due to widespread tissue necrosis. Leishmaniasis is also a common opportunistic infection of HIV, in addition rendering the distinction between viscerotropic species (causing VL) and dermatotropic species (causing CL) less valid [25]. *Leishmania* DNA has been shown to persist in humans after therapeutically achieved clinical resolution for *L*. (*Viannia*) in peripheral blood, lesions, mucosa and scars [26–29] of patients. Several studies provide evidence of the viability and infectivity of the parasite in some specimens isolated after clinical cure: Old world species (*L*. *infantum*) (syn. *L*. *chagasi*) [30,31]), as well as new world species (*L*. *(Viannia)* [26,32]), suggesting that persistence of parasites in both OWCL and NWCL might be common.

Fears surrounding involvement of tissues other than skin might indicate a fear of visceral leishmaniasis (VL), which is co-endemic in some countries such as Brazil, Tunisia and Morocco [33,34]. In addition, the vagueness of their descriptions indicates that there is a lack of knowledge.

Findings from the interviews supported the association of CL with social stigma, anxiety and depression. A Tunisian woman with diabetes who reported suicidal thoughts in case of amputation of her leg because of CL gives a good illustration of this aspect. Epidemiological studies conducted for OWCL have shown that around 50% of CL lesions are located in the face, and the visibility of lesions is considered an important risk factor for depression in dermatological conditions [35,36]. Other studies point out a significant association of CL with anxiety, depression and decreased QoL in patients [37–40]. Of great impact are ulcerative lesions and scarring on the faces of young women, exposing them to stigma and potentially affecting their marriage prospects [3,41,42].

### Implications for research practice: Recommendations based on patient-preferred outcomes

Information obtained on preferred outcomes was very rich, and we identified both generally-applicable and context-specific themes.

**Fig 3:**
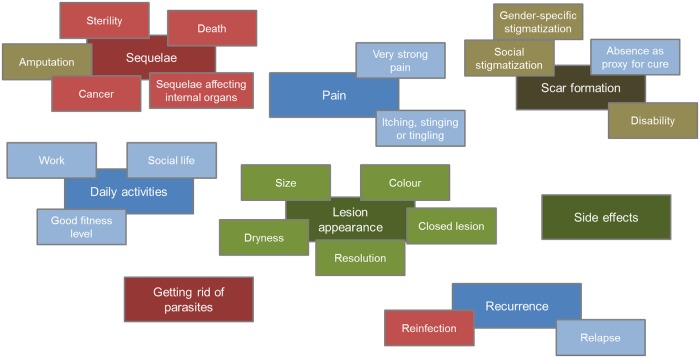
Reported outcome domains and outcomes by recommendations. Four groups distinguish outcomes according to the recommendations being given, these are discussed in more detail in the text. Green: Outcomes already included in clinical trials. Blue: Additional outcomes to be included in clinical trials. Brown/gold: Outcomes whose considerable psychological impact should be specifically addressed. Red: Outcomes where additional information should be given to patients.

Outcomes and outcome domains identified (Fig 2) were classified into four groups according to recommendations (Fig 3).

### Outcomes already included in clinical trials

The first group contains outcome domains and outcomes depicted in green, mostly concerning *Lesion appearance*, and also the most common *Side effects*. These are concepts corresponding to the clinical definition of cure with regards to lesion appearance already used and reported in clinical trials for CL interventions, that is, all inflammatory signs having disappeared, and epithelialization of ulcerative lesions.

### Additional outcomes to be included in clinical trials

The second group, depicted in blue, contains the domains *Daily activities*, *Pain* and *Recurrence* as well as the outcome *Absence as proxy for cure* in the domain *Scar formation*.

*Relapse*, contained in the domain *Recurrence*, is addressed in trials, but at varying time points [2–4,6]. In line with Olliaro et al. [11], we would recommend to consistently include six months (180 days) follow up to assess final cure. Taking into consideration potentially increased costs and the probability of loss to follow-up, this would be particularly recommended for phase IV and/or post-licensing surveillance studies.

We would argue that *Daily activities*, *Pain* and *Scar formation* (*via* its immediate impact on daily life) are Quality of Life (QoL)-related and could be measured, as a secondary outcome, using an appropriate instrument before treatment and at follow-up. The Dermatology Quality of Life Index (DLQI) [43] is an option to assess QoL-related aspects in clinical trials for CL. It contains questions relating to *Symptoms*, *Daily activities*, *Leisure activities*, *Work and school* and *Personal relationships*. The DLQI is standardized, used widely and has been translated into more than 90 languages. However, it is not validated for CL, nor consistently used, and does not include economic impact and access to health services, which are strongly related to QoL. A specific and comprehensive Cutaneous Leishmaniasis Impact Questionnaire (CLIQ) for assessment of the impact of CL on patients’ QoL was developed more recently by Galvao et al. [44] in Brazil, and has undergone initial validation.

As the absence of scars was considered a surrogate for cure, we would also recommend including outcomes assessing scar formation or prevention of scarring, in addition to its impact of QoL, in clinical trials, using e.g. the Vancouver Scar Scale [45] at six months after the end of treatment [46]. So far, prevention of scarring was only assessed in a small minority of OWCL trials [6].

A summary of recommendations can be found in Table 1.

**Table 1:** Recommendations for outcomes and outcome measures to be included in clinical trials.

Outcome domain	Patient-preferred outcome	Outcome (3,4,6)	Outcome measure
Recurrence	Relapse	Recurrence	Follow-up duration of 180 days (6 months)
Daily activities	Social life	QoL	QoL questionnaire, e.g. the DLQI [43] or the CLIQ [44] before treatment and at 6 months follow-up
	Work		
	Good fitness level		
Pain	Very strong pain		
	Itching, stinging or tingling		
Scar formation	Absence as proxy for cure		
		Degree of functional and aesthetic impairment and/or prevention of scarring	Scar assessment, e.g. Vancouver Scar Scale [45] at 6 months follow-up

Patient-preferred outcomes were mapped to outcome designations as listed in the reviews conducted by the Cochrane Collaboration [3,4,6] and outcome measures (measurement instruments) are suggested. Limitations of the DLQI [43] are discussed in the text.

### Outcomes whose considerable psychological impact should be specifically addressed

The third group, in brown and gold, contains outcomes broadly contained in the outcome domain *Scar formation*, such as *Social stigmatization* (and in particular *Gender-specific stigmatization* towards women) and *Disability*, as well as *Amputation* in the outcome domain *Sequelae*. *Amputation* is a rare, but possible, sequela in patients with comorbidities such as diabetes, or a sequela in those who develop MCL. Such patients form part of special populations and would not be included in routine trials.

These outcomes may have a considerable psychological impact on patients which should be consistently addressed. The DLQI [43] or the CLIQ [44] are options for assessment, given that they include impact on personal relationships and feelings as well as disabilities to a lesser extent. In addition, we would recommend informing patients, for example about treatment options for scars after the follow-up time, and if necessary, provide psychological support, in line with the considerable association of CL with anxiety and depression as discussed previously. These recommendations become even more important in the context of clinical care.

### Outcomes where additional information should be given to patients

The fourth group, in red, contains outcomes patients are afraid of, but which would not be meaningful outcomes for a clinical study. A number are grouped in the domain *Sequelae*, consisting of fears around *Sterility*, *Death*, *Cancer* and *Sequelae affecting internal organs*. These could be considered as misconceptions in relation to CL. *Getting rid of the parasite*, mostly expressed as the absence of parasites in the body, is an outcome patients would like to see. Whereas parasitological confirmation is strongly recommended for inclusion in trials, evidence of parasite persistence after clinical resolution of lesions, as discussed previously, renders parasitological cure most likely not a clinically meaningful outcome and would generate unnecessary concern for patients. *Reinfection*, in the domain *Recurrence*, was also found of concern to patients, but was not recommended for inclusion in trials.

We would suggest addressing outcomes in this group carefully by informing patients to clarify these issues and alleviate their fears, for example by inclusion of information in the *Patient Information* or *Informed Consent Declaration* for trials, through personal communication, or by using a ‘*Cutaneous Leishmaniasis Fact Sheet’* that could be given to patients at treatment onset in clinical care. Beyond volunteers in clinical trials, these findings demonstrate significant gaps in health education and support the need to account for these dimensions in future public health programs.

### Other findings

In general, reported outcomes were found to be quite uniform across regions. This section focusses on interpretation of two unexpected findings, or discrepant data: The fear of infertility as a potential sequela of CL treatment; and strong pain as a symptom of CL.

In Colombia CL is seen as an occupational disease, that is, a disease related to the military profession (L. López, personal communication). Five out of the six male Colombian patients mentioning fear of infertility were soldiers with the army. Infertility is not among the documented sequelae for either pentavalent antimonials or glucantime which was administered to patients interviewed [24]. This interesting finding could indicate a cultural notion related to a certain profession, or gender. As a significant life event, fathering a child can be integral to the production of a masculine identity [47], and the inability to do so could threaten that identity. The local PI pointed out the importance of the ability to father children in the Colombian context. She noted in addition that, since many of the soldiers were very young and undisciplined, their commanders were struggling to keep them in the military base while on treatment; the commanders therefore told them having sexual relations while on treatment could confer sterility. She concluded that sterility as possible sequel encountered in the interviews could be one of several variations of this rumour (L. López, personal communication). A Colombian anthropologist, working at the same site for her research on armed conflict and CL in Colombia, confirmed these findings (L. Pinto García, personal communication). In summary, the medically unfounded fears surrounding male infertility as possible sequel of CL treatment could be due to interrelated rumours in a very male, and male dominated, culture of *machismo* in the Colombian army, and there are indications that these rumours might have been produced on purpose.

Typically, CL lesions (nodules and ulcers) are not painful, unless secondary bacterial infection takes place—which is not uncommon, and may lead to pain and disability [3]. When discussing this strong emphasis on pain reported by patients in Burkina Faso with the local PI (M. Cissé), he noted that it occurred before traditional treatments were administered, instead of as a consequence, and saw it as part of the natural evolution of the disease. Results should be interpreted against the background that patients were from an emerging CL focus and have never experienced symptoms before (M. Cissé, personal communication). CL in new foci tends to be more aggressive [10]. This is in line with the high average number of lesions of patients in Burkina Faso (11, as compared to 3.4 of the entire study).

### Strengths and limitations of the study

This was a unique study involving partners from a number of different countries affected by CL, and engaging a range of patients whose voices have hitherto been unheard in CL research. It was an exploratory study, aiming to elicit the views of a patient population as large and diverse as possible. As a qualitative study, it is not intended to be representative of a given study population, however most key themes were remarkably consistent across the different countries and patients groups despite cultural and disease heterogeneities.

Interviews were being conducted in a range of local contexts and six different languages (Arabic, Dioula, Farsi, Mooré, Portuguese and Spanish). The analysis of pooled interviews, conducted by two researchers and greatly relying on notes taken during the interviews and discussions with the local PIs was able to ensure consistency and take the cultural context into consideration.

We did not set a maximum time limit between the end of treatment and the time point when interviews would take place, in line with our maximum variation sampling approach. This aspect was considered important in order to allow participants to reflect on a long duration of experiences, such as those related to scars. The topic guide was designed as being as conducive as possible to participants speaking freely, in order to also recall distant memories. At the same time, participants are expected to have had a selective memory recall during the interviews.

By design, there was a focus on implications for clinical trial design; therefore we did not include patients who would not normally be enrolled, e.g. patients with comorbidities or more severe disease manifestations.

Children were also excluded, despite the fact that they form a significant part of the patient population in some countries such as Iran. Interviewing children requires a skillset and precautions that were beyond the resources of our study [48]. Future studies should focus on these important populations because most cases of OWCL are among children in old foci and the co-existence of CL and chronic diseases, such as diabetes among elderly, in emerging foci.

### Further research

When the study findings are compared to the outcomes traditionally included in trials, we see gaps, which overlap with those already discussed by three Cochrane Collaboration reviews [3,4,6]: outcomes related to the degree of functional and aesthetic impairment (which we suggest correspond to the outcomes contained in the domain *Sequels* or *Disability* associated with scars in our study), prevention of scarring (outcomes domain *Scar formation*) and QoL (outcomes contained in the domains *Daily activities* or *Pain*).

Validated measurement instruments for dermatologic conditions would allow for inclusion and measurement of these endpoints in clinical trials. Current limitations of the DLQI can be overcome by development of an instrument validated for NTDs, or specifically for CL, such as the CLIQ. The interviews also pointed out some fears and misconceptions surrounding CL, and a need for better informing and counselling patients.

The next step in our project is now to bring these results to the attention of the broader health provider, drug developer and researcher communities, and specialists in ethics so that they can be taken into account when designing new treatments and clinical trials. We propose a Delphi consensus survey including a large number of HCPs and researchers following the methodology developed by COMET for COS development [19,49]. The survey questionnaire will combine outcomes reported in the literature with outcomes reported during this study and recommended for inclusion in clinical trials. The aim of this study is the development of a COS for CL, consisting of a consensus set of proposed outcomes and their measurement instruments recommended for inclusion in future clinical trials, therefore working towards harmonization of clinical trial methodology and including patients’ voices (11).

## Conclusions

This study shows how otherwise disempowered patients suffering from a neglected tropical disease can be effectively involved in research, providing valuable insights into desired outcomes, and be given a voice in designing both new medications and treatment approaches and the way these are tested in clinical trials. We believe that combining expert [10,11] and patient consultation, if properly acted upon, can promote better trials and lead to more adapted interventions. With few notable exceptions, such as the OMERACT initiative for rheumatologic conditions [50] these steps as seldom taken, especially when it comes to poverty-related diseases affecting disproportionally disenfranchised populations. The work we did in CL shows that this is possible, and can be seen as an exemplar which can be applied to other similar situations.

The proposed development of a COS for CL would be a further step towards including patients’ opinions into the harmonization of clinical trial methodology [11].

## Supporting information

S1 File An overview of study sites and demographic and clinical characteristics of patients enrolled in the study. (DOC)
