# Supplementary material for: Patients’ preferences of cutaneous leishmaniasis treatment outcomes: Findings from an international qualitative study
Source: PLoS Negl Trop Dis. 2020 Feb 24;14(2):e0007996. doi: 10.1371/journal.pntd.0007996 (PMC7058360; doi:10.1371/journal.pntd.0007996)
Supplement: S1 File — (DOC) [file pntd.0007996.s001.doc]

## Supporting information 1: Patient characteristics

|  | **Brazil** | **Burkina Faso** | **Colombia** | **Colombia** | **Iran** | **Morocco** | **Peru** | **Tunisia** | **Overall** |
| --- | --- | --- | --- | --- | --- | --- | --- | --- | --- |
| **Institution/Study site** | Centro de Pesquisa René Rachou (CPqRR), Fundação Oswaldo Cruz (FIOCRUZ) Minas Gerais | Centre MURAZ, Bobo-Dioulasso | Centro Internacional de Entrenamiento de Investigaciones Médicas (CIDEIM), Cali | Programa de Estudio y Control de Enfermedades Tropicales (PECET), Medellín | Molecular Dermatology Research Center, Shiraz University of Medical Sciences, Shiraz | National School of Public Health, Rabat | Instituto de Medicina Tropical Alexander von Humboldt, Universidad Peruana Cayetano Heredia, Lima | Institut Pasteur de Tunis, Tunis |  |
| **Area where the study is conducted** | Belo Horizonte | Bobo-Dioulasso and Ouagadougou | Cali and Tumaco | Leishmaniasis Recovery Center, Boyacá | Shiraz and vicinity | Sefrou, Moulay Yacoub | Lima, San Martin, Cusco | Sidi-Bouzid and Gafsa |  |
| ***Leishmania* species** | *L. (Viannia) braziliensis* | *L. major* | *L. (Viannia) panamensis* | *L. (Viannia) braziliensis, L. (Viannia) panamensis* | *L. major, L. tropica* | *L.tropica* | *L. (Viannia) braziliensis and L. (Viannia) peruviana* | *L. major* | L. b. 24%, L. b. or L. pe. 9%, L. m. 14%, L. m. or L. t. 26%, L. pa. 16%, L. t. 11% |
| **Number of patients** | 10 | 10 | 10 | 10 | 10 | 8 | 7 | 9 | 74 |
| **Age range of patients (mean, median) in years** | 19-71 (42, 41) | 24-73 (52, 56) | 18-52 (34, 34) | 20-32 (25, 25) | 21-63 (38, 33) | 23-57 (42, 50) | 25-68 (42, 37) | 27-65 (39, 37) | 18-73 (39, 35) |
| **Gender ratio (F:M)** | 50:50 | 40:60 | 20:80 | 0:100 | 50:50 | 63:37 | 29:71 | 56:44 | 38:62 |
| **Average number of lesions (mean, median)** | 1-1 (1, 1) | 1-56 (11, 6) | 1-4 (1.7, 1) | 1-5 (2.3, 1) | 1-5 (1.7, 1) | 1-6 (2.5, 2) | 1-3 (2.1,3) | 1-5 (3.2,3) | 1-56 (3.4, 1) |
| **Treatment status in % (diagnosed:under treatment:completed:untreated)** | 0:40:60:0 | 0:0:90:10 | 20:40:40:0 | 20:30:50:0 | 0:70:30:0 | 0:25:75:0 | 0:100:0:0 | 0:89:11:0 | 5:47:46:1 |

Notes: L.b….L. *(Viannia) braziliensis*, L.m….L. *major*, L.pa….L. *(Viannia) panamensis*, L.pe…L. *(Viannia) peruviana*, L.t….L. tropica
